# EFFECTS OF COVID-19 DISINFECTION RECOMMENDATIONS ON MICROBIAL ENVIRONMENT CONTAMINATION: FOCUS ON EMERGENCY PHYSICIANS' STETHOSCOPES AND SMARTPHONES

**DOI:** 10.13075/ijomeh.1896.02444

**Published:** 2025

**Authors:** Prabakar Vaittinada Ayar, Angèle Cassagne, Pradeebane Vaittinada Ayar, Bruno Ferrao, Simone Nerome, Matthieu Gay

**Affiliations:** 1 Beaujon Hospital AP-HP, Emergency Department, Clichy, France; 2 INSERM UMR-S942, MASCOTT, Paris, France; 3 University of Paris, Paris, France; 4 Paris-Saclay University, Laboratory of Climate and Environmental Sciences (LSCE-IPSL) CNRS/CEA/UVSQ, UMR8212, Gif-sur-Yvette, France; 5 Beaujon Hospital AP-HP, Bacteriology – Hygiene – Virology – Parasitology Department, Clichy, France

**Keywords:** hygiene, disinfection, bacterial contamination, COVID-19, emergency department, stethoscopes and smartphone contamination

## Abstract

**Objectives:**

The coronavirus disease 2019 (COVID-19) has considerably changed the game in the field of hygiene. The aim of the study was to compare microbiological colonization present on the emergency physicians' stethoscopes and smartphones before and after the outbreak of COVID-19.

**Material and Methods:**

This was a prospective cohort study in 1 academic hospitals' emergency department. A microbiological analysis was conducted on the emergency doctors' stethoscopes and smartphones for a month in 2018 and 2021. Analysis concerned stethoscopes diaphragms and the most used surface of the cellphones screen around to the main button. The authors used a solid growth medium irradiated Count-Tact^®^ 3P agar (CT3P) (BioMerieux, Lyon, France) for collecting samples. Results were obtained after 5 days of growth at 30°C to collect all the saprophytes environmental flora.

**Results:**

A total of 27 doctors were included in 2018 and 30 doctors in 2021. Stethoscope diaphragm contamination was very high in both period with a geometric mean (GM) without difference before and after COVID respectively, GM = 68 colony-forming unit (cfu) per 25 cm^2^ (95% CI: 50–94 cfu/25 cm^2^) vs. 68 cfu/25 cm^2^ (95% CI: 44–105 cfu/25 cm^2^), p > 0.05. Smartphones were cleaner than stethoscopes with a GM <50 cfu/25 cm^2^ without significant difference between 2 periods, respectively GM = 45 cfu/25 cm^2^ (95% CI: 34–59 cfu/25 cm^2^) vs. 31 cfu/25 cm^2^ (95% CI: 20–48 cfu/25 cm^2^), p > 0.05.

**Conclusions:**

The study shows an urgent need to regularly inform of the hygiene of the medical tools and COVID-19 does not really bring improvements in the matter. Particularly in emergency department, where physicians examine several patients per day and can possibly transmit pathogens.

## INTRODUCTION

Microbiological risks associated with environmental contamination remain a significant public health concern, particularly as contaminated surfaces are a known source of healthcare-associated infections [1]. A European annual report published a decade ago estimated >2.6 million cases of hospital-acquired infections (HAIs) annually, with nosocomial diseases responsible for approx. 37 000 deaths/year [2]. In France alone, ≤750 000 nosocomial cases are reported each year [3]. Medical and non-medical equipment, including stethoscopes, otoscopes, reflex hammers, smartphones, and computers, have been shown to harbor significant bacterial colonization [4]. In recent years, the emergence of highly resistant bacteria has been documented, although the global incidence of multidrug-resistant bacteria has remained relatively stable [5,6].

Medical devices frequently used by physicians, such as stethoscopes, serve as reservoirs for pathogens, including methicillin-resistant *Staphylococcus aureus* (MRSA), vancomycin-resistant *Enterococci* (VRE), *Clostridioides difficile*, *Pseudomonas aeruginosa*, and *Klebsiella* species [7]. The contamination level of stethoscopes has been correlated with the contamination level of healthcare workers' hands [8]. This “third hand” of physicians can act as a vector for infections, even when proper hand hygiene is performed. During procedures such as intubation, urinary catheter placement, or physical examinations, stethoscopes can transfer pathogens to hands or gloves, potentially leading to HAIs, ventilator-associated pneumonia, gastrointestinal infections, urinary tract infections, or surgical site infections [9]. Serious cases of *Clostridioides difficile* infections have also been reported after the use of contaminated stethoscopes [10]. Similarly, smartphones, which have become indispensable tools in modern medical practice, pose a comparable risk [11].

The disinfection of medical devices is critical for the primary prevention of HAIs and the containment of highly resistant bacteria, which remains a major focus in hospital infection control programs [12,13]. The 3 principal strategies for infection prevention include hand disinfection, the use of alcohol-based solutions, and the decontamination of medical equipment [14]. French hospitals have implemented comprehensive infection control measures, including the establishment of nosocomial infection control committees (Comité de Lutte contre les Infections Nosocomiales – CLIN) and local infection control teams (Équipe Mobile d'Hygiène). Guidelines issued by the Ministry of Health and Solidarity provide recommendations and best practices for maintaining hygiene standards [15].

In emergency departments, where physicians work under considerable time pressure, the risk of microbiological cross-transmission is heightened. Physicians often move rapidly between patients, using the same stethoscope without adequate time for disinfection. For instance, one hand may palpate the abdomen of a surgical patient while the other dials a radiologist to expedite a CT scan. Such practices increase the likelihood of transferring highly resistant pathogens [16–18].

The emergence of coronavirus disease 2019 (COVID-19) has drastically reshaped hygiene practices. The pandemic heightened awareness of environmental disinfection, leading hospitals to adopt mass use of both alcoholic and non-alcoholic disinfectants to prevent COVID-19 transmission [19]. Standard hygiene protocols for physicians and hospital staff were reinforced [20], and recent reviews have summarized scientific evidence supporting environmental cleaning to curb COVID-19 transmission [21].

While these measures have proven effective in managing the pandemic [12], their impact on bacterial and fungal colonization has not been well-documented. The aim of this study is to compare the microbiological colonization of emergency physicians' stethoscopes and smartphones before and after the COVID-19 outbreak.

## MATERIAL AND METHODS

This prospective observational study was conducted in the emergency department of an academic hospital (Hôpital Beaujon, Assistance Publique–Hôpitaux de Paris, Clichy, France). Microbiological analyses were performed on the stethoscopes and smartphones of emergency physicians over the course of 1 month, as part of departmental hygiene assessments. The first analysis was conducted prior to the COVID-19 pandemic, February–March 2018, with data collected for potential future comparison. A second analysis was performed in May–June 2021, immediately following the third local wave of the COVID-19 outbreak. Samples were taken from the diaphragms of stethoscopes (further referred as “stethoscopes”) and the most frequently used area of smartphone screens, around the main button (further referred as “smartphones”). A single sample was collected from each surface using direct contact with a solid growth medium. The same operators and analytical techniques were used for both time periods.

### Study protocol

Participants were not informed about the ongoing study to minimize behavioral changes. Sampling times and dates were not pre-scheduled, and participants were unable to clean their equipment prior to sample collection. Each participant was directed to a dedicated room for sampling. Samples were then transported to the hospital's microbiology laboratory for analysis. Limited participant data, such as sex and physician status (senior or intern), were collected, and all results were anonymized. Participation in the study was voluntary, and participants could withdraw without any repercussions. No penalties or rewards were associated with the contamination levels of their items.

### Sample collection and analysis

Samples were collected using irradiated Count-Tact^®^ 3P agar (CT3P) plates (BioMerieux, Lyon, France), which have an internal diameter of 55 mm. The growth medium consisted of casein peptone (bovine) (15.0 g/l), soy peptone (5.0 g/l), yeast extract (6.0 g/l), sodium chloride (5.0 g/l), sodium pyruvate (2.0 g/l), soy lecithin (0.7 g/l), polysorbate 80 (5.0 g/l), sodium thiosulfate pentahydrate (0.05 g/l), L-histidine (1.0 g/l), agar (20.5 g/l), and purified water. Samples were collected by the infection control physician directly in the emergency department. The samples were incubated for 5 days at 30°C to allow for the growth of saprophytic environmental microbiota. Colonies were counted and reported as colony-forming units (cfu) per 25 cm^2^ (cfu/25 cm^2^). The detection limit was set at 150 cfu/25 cm^2^. A threshold of 50 cfu/25 cm^2^ (equivalent to 2 cfu/cm^2^) was established as the theoretical target value for emergency departments, in accordance with French Normalization Agency standards [22], which define an acceptable contamination level of <5 cfu/cm^2^ or <20 cfu on the stethoscope membrane. Samples exceeding this threshold were classified as “highly contaminated” and were considered likely to contain ≥1 pathogens. Due to the high bacterial load, identification of specific pathogens was challenging with routine methods. Microbial colonies were identified using a combination of conventional biochemical methods. To isolate and differentiate bacterial species, the following selective and differential culture media were used: MacConkey agar, mannitol salt agar, cetrimide agar, blood agar, sabouraud dextrose agar. Microorganisms were identified by matrix-assisted laser desorption/ionization time-of-flight (MALDI-TOF) mass spectrometry.

The biochemical tests performed included Gram staining to determine bacterial morphology and classification, as well as oxidase and catalase tests to differentiate between bacterial species. Additional tests included citrate utilization, arabinose, and lactose fermentation, with cultures grown on MacConkey agar to assess the ability of Gram-negative bacteria to ferment lactose. Selective and differential media were used where appropriate to aid in pathogen identification.

For MALDI-TOF mass spectrometry analysis, colonies were directly spotted onto a steel target plate and overlaid with a matrix solution composed of α-cyano-4-hydroxycinnamic acid (CHCA) in 50% acetonitrile and 2.5% trifluoroacetic acid. After air drying, the plate was inserted into the MALDI-TOF Biotyper (Microflex Bruker Daltonics/BioTyper^™^, v. 2.0, Wissembourg, France) for protein spectrum acquisition.

### Ethics statement

This study was conducted in compliance with the Declaration of Helsinki. Physicians involved in the study were informed of its objectives, and written consent was obtained from all participants. The study was approved by the French National Commission for Information Technology and Civil Liberties (CNIL) (No. 2233058) and the ethics committee of the Hôpitaux Universitaires Paris Nord Val de Seine, France.

### Statistical analysis

Data were expressed as geometric means (GMs) with 95% confidence intervals (95% CI). Analyses were performed using analysis of variance, with a significance threshold of p < 0.05. Categorical variables are presented as numbers (percentages). Normality was assessed using the Shapiro–Wilk test. Group characteristics were compared using t-tests for normally distributed continuous variables, Mann–Whitney U tests for non-normally distributed continuous variables, and χ^2^ tests for categorical variables. Statistical analyses were conducted using R software (R Foundation for Statistical Computing, Vienna, Austria). A 2-sided p-value of <0.05 was considered statistically significant.

## RESULTS

A total of 27 doctors were included in the 2018 pre-COVID-19 analysis. Among them, 11 were male (41%), and 9 were residents (33%). Following the third local wave of the COVID-19 pandemic in 2021, samples were collected from 30 doctors, of whom 9 were male (30%) and 11 were residents (37%). Screening covered 96% of the department's physicians, with only 1 doctor excluded during each study period.

### Bacterial contamination

Stethoscope diaphragms exhibited high levels of contamination in both study periods, with a GMs >50 cfu/25 cm^2^ (Table 1). There was no statistically significant difference in stethoscope contamination levels before and after the pandemic (p > 0.05), with respective GMs of 68 cfu/25 cm^2^ (95% CI: 50–94 cfu/25 cm^2^) in 2018 and 68 cfu/25 cm^2^ (95% CI: 44–105 cfu/25 cm^2^) in 2021.

**Table 1.** Contamination of smartphones and stethoscopes used by emergency medical services personnel pre-COVID-19 (N = 27) and post-COVID-19 (N = 30), Beaujon Hospital, Clichy, France, February–March 2018 and May–June 2021

Variable	Contamination [cfu/25 cm^2^]																															Devices with >50 cfu/25 cm^2^ [n]
	level																														GM (95% CI)	
Before COVID-19																																
position	S	S	S	S	S	S	S	S	I	I	S	S	I	I	I	I	S	S	I	S	I	S	S	I	S	S	S					
device																																
stethoscope	10	100	45	100	50	60	80	46	63	150	15	150	150	98	150	150	55	80	150	91	100	65	13	150	150	9	100				68.5 (50–94)	20
smartphone	90	100	12	30	32	60	40	39	35	36	31	150	31	42	70	47	150	67	100	36	42	49	130	33	29	32	5				45 (34–59)	9
After COVID-19																																
position	S	S	S	I	I	S	S	I	I	S	I	S	S	S	S	S	I	I	S	I	I	I	I	S	I	S	S	S	S	S		
device																																
stethoscope	27	75	18	150	150	150	70	150	150	50	82	150	150	150	150	150	150	81	96	55	1	64	9	150	150	150	66	89	4	53	68 (44–105)	27
smartphone	2	150	10	2	54	6	70	42	29	49	13	60	12	61	61	66	150	40	150	9	47	115	121	93	7	10	50	13	12	78	31 (20–48)	13

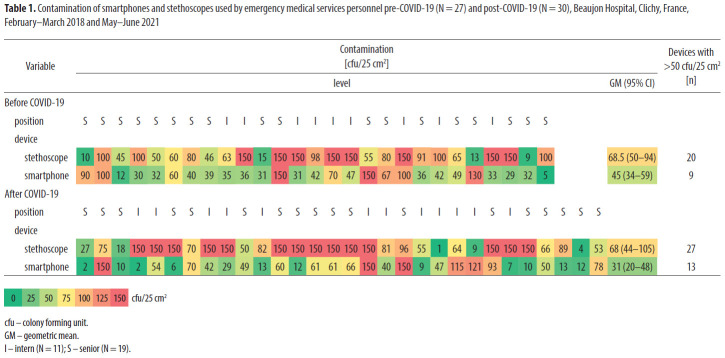

0 25 50 75 100 125 150 cfu/25 cm^2^

cfu – colony forming unit.

GM – geometric mean.

I – intern (N = 11); S – senior (N = 19).

**Figure 1.**
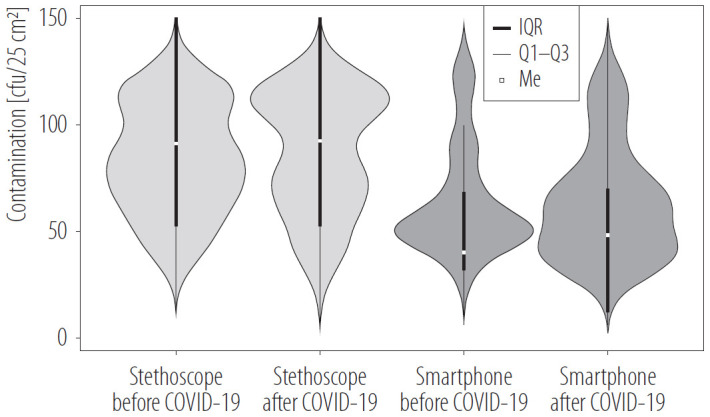
Contamination evolution of pre-COVID-19 (N = 27) and post-COVID-19 (N = 30) emergency physician stethoscopes and smartphones used by emergency medical services personnel, Beaujon Hospital, Clichy, France, February–March 2018 and May–June 2021

Smartphones, in contrast, demonstrated lower contamination levels than stethoscopes, with GMs <50 cfu/25 cm^2^ in both periods (Table 1). No statistically significant difference was observed between the 2 periods (p > 0.05), with GMs of 45 cfu/25 cm^2^ (95% CI: 34–59 cfu/25 cm^2^) in 2018 and 31 cfu/25 cm^2^ (95% CI: 20–48 cfu/25 cm^2^) in 2021. However, a notable reduction in bacterial load on smartphones was observed, with contamination levels decreasing by 32% post-pandemic (Figure 1). Stethoscope contamination levels showed no such reduction.

### Pathogen identification

In 2018, contamination with methicillin-susceptible *Staphylococcus aureus* (MSSA) was identified in 2 samples (1 stethoscope and 1 smartphone). In 2021, 5 samples were contaminated with pathogenic bacteria, including 2 stethoscopes and 3 smartphones. Identified pathogens included *Pseudomonas aeruginosa* (N = 2), *Acinetobacter johnsonii* (N = 2), and MSSA (N = 1).

### Fungal contamination

In 2018, fungal contamination with *Aspergillus* species was observed in 2 samples: 1 stethoscope (*A. niger*) and 1 smartphone (*Aspergillus* sp.). By 2021, fungal contamination increased to 5 samples, comprising 2 stethoscopes and 3 smartphones, all contaminated with *Aspergillus* sp.

## DISCUSSION

This monocentric study highlights the significant bacterial and fungal contamination of stethoscopes and smartphones, consistent with their recognized roles as bacterial reservoirs [8–10]. Guidelines emphasize the necessity of regular disinfection of non-medical equipment, with stethoscopes prioritized as critical tools for patient care [23].

### Effects of COVID-19 pandemic on stethoscope and smartphone disinfection

Despite the heightened awareness of hygiene during the COVID-19 pandemic, the authors' findings indicate persistent high contamination levels on stethoscopes and smartphones. While smartphones exhibited a 32% reduction in bacterial load post-pandemic, stethoscope contamination levels remained unchanged. These results suggest that routine disinfection remains insufficient, particularly in high-pressure environments like emergency departments, where adherence to hygiene protocols is often suboptimal [18,24]. Disinfection is not a routine practice, despite the proven efficiency of many methods [25], and this challenge appears to persist across other hospital units as well [26]. The COVID-19 pandemic initially raised awareness of surface hygiene due to concerns about SARS-CoV-2 survival on surfaces [27]. Numerous infection control measures were implemented, including awareness campaigns by infection control teams. However, the authors' findings suggest that these measures did not significantly alter daily hygiene practices for personal medical devices. The continued presence of fungi on stethoscopes and smartphones in both study periods indicates a lack of regular cleaning shortly before use [28].

### Vector of healthcare-associated infections

The present study demonstrated that both smartphones and stethoscopes were contaminated with pathogens, including bacteria and fungi. To prevent the spread of nosocomial infections, the use of patient-specific stethoscopes is recommended. This practice is already standard for patients colonized with highly resistant bacteria, such as glycopeptide-resistant *Enterococcus faecium* and carbapenemase-producing *Enterobacteriaceae*. However, implementing similar measures for smartphones is impractical. Mobile phones have been recognized as potential vectors for transmitting pathogenic microorganisms since at least 2007 [29]. Over the last decade, smartphones with touchscreens have become increasingly efficient and indispensable tools for healthcare professionals, helping save time in patient care [30]. Their close proximity to patients and frequent handling heighten the risk of contamination with pathogens [31]. These microorganisms can contribute to nosocomial infections [32]. One study suggested that regular disinfection might prevent the transmission of COVID-19 to the family members of healthcare professionals [33]. However, there is limited evidence supporting the benefits of disinfection in reducing SARS-CoV-2 transmission or its direct impact on bacterial reservoirs, as previously observed. The COVID-19 pandemic has increased awareness of the importance of mobile phone hygiene [34]. Similarly, stethoscopes are frequently colonized by pathogenic bacteria. The most common contaminants include *Staphylococcus* species such as MRSA, coagulase-negative *Staphylococcus*, and *Clostridioides difficile*, as well as *Pseudomonas aeruginosa* and VRE [9]. Longtin et al. [8] found that the contamination levels of stethoscope diaphragms were comparable to those of physicians' fingertips, with the diaphragm being the second most highly contaminated site after the fingertips. This high contamination level is likely due to the frequent use of stethoscopes by multiple patients and doctors, making them more prone to cross-contamination than smartphones.

The study highlighted the need for consistent disinfection practices and served as a reminder to participating physicians about the importance of equipment hygiene. Interestingly, during the initial wave of COVID-19, the introduction of vaccination and a possible sense of protection among healthcare professionals may have contributed to a decline in adherence to good hygiene practices.

### Alternative of disinfection

When a patient was suspected of having COVID-19 in the authors' hospital, dedicated protective devices and stethoscopes were used during examinations. These devices were either discarded after use or thoroughly disinfected. The challenges associated with maintaining cleanliness for personal stethoscopes highlight the need to explore alternative solutions. One such approach could involve equipping each consultation room with dedicated stethoscopes, which could mitigate contamination and improve infection control. Innovative solutions, such as aseptic stethoscope barriers, could significantly reduce pathogen transmission and deserve serious consideration [35].

A recent review demonstrated that these barriers are superior to traditional cleaning practices using chemical agents, provided they are single-use, disposable, applied in a touch-free manner, impervious to pathogens, ensure aseptic patient contact, and are acoustically invisible [36]. Additionally, stethoscope surfaces made from antimicrobial copper alloys have consistently been shown to harbor fewer bacteria, making them another viable option [25,37]. Another promising physical method of disinfection involves the use of ultraviolet (UV) light. Wearable devices emitting UV-C light through light-emitting diode (LED) technology have proven effective against common microorganisms associated with healthcare-associated infections [25,38]. These devices can efficiently disinfect stethoscope membranes, even when they are heavily contaminated. In real-world environments, studies have reported a 94.8% reduction in cfus (95% CI: 91.3–97.7%) with the use of UV-C devices [39].

The main limitation of this study lies in the technical challenges of identifying all pathogens. Given the significance of surface contamination, accurately identifying all microorganisms using routine methods proved difficult. This limitation is important, as it may affect the awareness and implementation of appropriate hygiene measures. While this study provides valuable insights into the microbial profile of healthcare-associated infections, the absence of antimicrobial susceptibility testing (AST) for the isolated pathogens represents a limitation. This omission restricts the ability to provide direct evidence of resistance patterns within this cohort. However, these findings contribute to the broader understanding of pathogen prevalence in similar settings and underscore the importance of incorporating AST in future research to guide empiric antimicrobial therapy.

## CONCLUSIONS

The study highlights the urgent need for regular reinforcement of hygiene practices for medical tools, particularly as the COVID-19 pandemic has not significantly improved this aspect. In the emergency department, where physicians examine multiple patients daily, the risk of pathogen transmission remains high. Stethoscopes, due to their shared use between patients and doctors, present a greater likelihood of cross-contamination compared to smartphones. Given these findings, there is a clear need to reconsider departmental protocols and explore the implementation of new barrier devices to reduce the risk of healthcare-associated infections.
